# Whole-genome characterization of hemolytic uremic syndrome-causing Shiga toxin-producing *Escherichia coli* in Sweden

**DOI:** 10.1080/21505594.2021.1922010

**Published:** 2021-05-03

**Authors:** Ying Hua, Milan Chromek, Anne Frykman, Cecilia Jernberg, Valya Georgieva, Sverker Hansson, Ji Zhang, Ann Katrine Marits, Chengsong Wan, Andreas Matussek, Xiangning Bai

**Affiliations:** a Department of Microbiology, School of Public Health, Southern Medical University, Guangzhou, China; b Division of Clinical Microbiology, Department of Laboratory Medicine, Karolinska Institutet, Huddinge, Sweden; c Division of Pediatrics, Department of Clinical Science, Intervention and Technology, Karolinska Institutet and Karolinska University Hospital, Stockholm, Sweden; d Queen Silvia Children’s Hospital, Sahlgrenska University Hospital, Gothenburg, Sweden; e Department of Pediatrics, Institute of Clinical Sciences, Sahlgrenska Academy, University of Gothenburg, Gothenburg, Sweden; f The Public Health Agency of Sweden, Solna, Sweden; g Molecular Epidemiology and Public Health Laboratory, School of Veterinary Sciences, Massey University, Palmerston North, New Zealand; h Laboratory Medicine, Jönköping Region County, Department of Clinical and Experimental Medicine, Linköping University, Jönköping, Sweden; i Oslo University Hospital, Oslo, Norway; j Division of Laboratory Medicine, Institute of Clinical Medicine, University of Oslo, Norway; k Division of Infectious Diseases, Department of Medicine Huddinge, Karolinska Institutet, Huddinge, Sweden; l State Key Laboratory of Infectious Disease Prevention and Control, National Institute for Communicable Disease Control and Prevention, Chinese Center for Disease Control and Prevention, Beijing, China

**Keywords:** Shiga toxin-producing *Escherichia coli*, hemolytic uremic syndrome, clinical outcomes, O157:H7, virulence genes, whole-genome sequencing

## Abstract

Shiga toxin-producing *Escherichia coli*, a foodborne bacterial pathogen, has been linked to a broad spectrum of clinical outcomes ranging from asymptomatic carriage to fatal hemolytic uremic syndrome (HUS). Here, we collected clinical data and STEC strains from HUS patients from 1994 through 2018, whole-genome sequencing was performed to molecularly characterize HUS-associated STEC strains, statistical analysis was conducted to identify bacterial genetic factors associated with severe outcomes in HUS patients. O157:H7 was the most predominant serotype (57%) among 54 HUS-associated STEC strains, followed by O121:H19 (19%) and O26:H11 (7%). Notably, some non-predominant serotypes such as O59:H17 (2%) and O109:H21 (2%) also caused HUS. All O157:H7 strains with one exception belonged to clade 8. During follow-up at a median of 4 years, 41% of the patients had renal sequelae. Fifty-nine virulence genes were found to be statistically associated with severe renal sequelae, these genes encoded type II and type III secretion system effectors, chaperones, and other factors. Notably, virulence genes associated with severe clinical outcomes were significantly more prevalent in O157:H7 strains. In contrast, genes related to mild symptoms were evenly distributed across all serotypes. The whole-genome phylogeny indicated high genomic diversity among HUS-STEC strains. No distinct cluster was found between HUS and non-HUS STEC strains. The current study showed that O157:H7 remains the main cause of STEC-associated HUS, despite the rising importance of other non-O157 serotypes. Besides, O157:H7 is associated with severe renal sequelae in the follow-up, which could be a risk factor for long-term prognosis in HUS patients.

## Introduction

Shiga toxin-producing *E. coli* (STEC) infections can cause a spectrum of clinical manifestations ranging from asymptomatic carriage to the life-threatening complication hemolytic uremic syndrome (HUS), which is characterized by microangiopathic hemolytic anemia, thrombocytopenia, and acute kidney injury (AKI) [1,2]. Most HUS cases are caused by STEC infection, whereas atypical HUS (aHUS) is associated with gene mutations or autoantibodies, which results in complement dysregulation [3]. It is estimated that 6–25% of patients with STEC-related hemorrhagic colitis develop HUS, among which, up to 50% of patients need dialysis during the acute phase [4]. STEC associated-HUS has been described in all age groups but most susceptible to very young children below five years and the elderly [5]. Serotype O157:H7 has been implicated worldwide as the major cause of post-diarrhea HUS either during STEC outbreaks or in sporadic cases [1]. Previous study showed that patients with HUS was significantly more likely to be infected with O157:H7 strains belonging to clade 8 lineage, which might acquire critical factors leading to more severe disease [6]. It is estimated that up to 15% of STEC-infected children below ten years developed HUS [1]. Nevertheless, non-O157:H7 STEC strains, especially those of serogroups O26, O103, O111, and O145, are variably present as causes of HUS and diarrhea throughout Europe [7–9] and other countries [10,11]. In 2011, an *E. coli* O104: H4 outbreak resulted in 4,321 confirmed cases, 852 HUS, and 54 deaths in Europe [12–14], highlighting the clinical importance of non-O157 serotypes.

The pathophysiology of HUS is thought to be due to the production of bacteriophage-encoded Shiga toxin (Stx), which causes endothelial damage and activates the complement cascade, leading to thrombotic microangiopathy that principally affects the kidney. It may also involve the central nervous system (CNS), heart, liver, pancreas, and hematopoietic cells [3,4,15]. Stx comprises two immunologically distinct toxin types, Stx1 and Stx2, which share 56% amino acid sequence similarity [16]. Strains producing Stx2, especially Stx2a, Stx2c, or Stx2d subtypes, show a stronger association with the development of HUS than other subtypes [17,18]. Aside from *stx*, additional virulence factors also contribute to the pathological process. For instance, *eae*-encoded intimin, is an important “effacement/adherence” factor in intestinal colonization [19,20], which can induce a strong antibody response in HUS patients [21]. Several other potential adherence factors, such as ToxB, Saa, Iha, Efa1, and Paa, have also been described in HUS-STEC strains [22,23]. However, there is no single or combination of genetic markers that can predict the potential of STEC strains to cause HUS, as various bacterial and host-related factors contribute to the pathogenicity. The correlation between bacterial genetic factors and clinical symptoms is not fully understood.

The prognosis of HUS has improved over time due to the advances in intensive care and dialysis. However, HUS in children is still a significant cause of mortality, which varies between 1% and 5% of cases [24]. In the acute phase, nearly 50% of the STEC-HUS patients need dialysis [25]. In the long term, 15–30% develop proteinuria, 5–15% hypertension, 9–18% decreased kidney function, and 3% even end-stage renal disease [25]. Prolonged dialysis and the long duration of oligo-anuria have been described as the most significant risk factors for an unfavorable long-term outcome. Besides, the presence of neurological symptoms, high white blood cell counts, high hematocrit levels, have also been suggested as risk factors for poor prognosis [25–27]. However, there is no curative treatment for STEC-HUS yet. Antibiotic therapy during the acute gut STEC infection is considered contraindicated as it boosts the release of Stx and stimulates the absorption of toxin, which ultimately increases the risk of HUS [28].

In Sweden, human STEC O157 cases have been mandatory to report since 1996 and all serotypes since July 2004 [29]. Most STEC infections were sporadic cases or smaller family outbreaks, although a few community outbreaks have been described [30–32]. In 2005, a large O157 outbreak occurred on the west coast of Sweden, 135 cases were recorded totally, including 11 cases of HUS [29]. However, limited study had described molecular characteristic of HUS-causing STECs of all serotypes in Sweden so far. Comprehensive analysis is needed to unveil the entire molecular picture of HUS-STEC strains, and to identify bacterial genetic factors associated with HUS clinical outcomes. In the current study, we report STEC associated-HUS cases treated in Gothenburg and Stockholm in Sweden from 1994 through 2018. By whole-genome sequencing-based molecular characterization and comparative genomic analysis, we attempt to ascertain molecular features of highly pathogenic HUS-associated STEC strains, and to identify bacterial genetic markers for severe outcomes in HUS patients.

## Materials and Methods

### Case definition, collection of isolates and clinical data

HUS was characterized by three primary symptoms: thrombocytopenia, microangiopathic hemolytic anemia, and acute kidney injury [33]. The HUS-associated STEC isolates were collected from routine STEC diagnostics. Clinical data were collected by reviewing the patients’ medical records. The metadata are shown in Table S1. Two isolates were originated from the same patient who sought medical treatment in two cities.

We investigated six clinical parameters during the acute phase of HUS. For each parameter, we divided patients into two groups: severe and mild. Hemoglobin concentration (Hb) ≤65 g/L, leukocytes (LPK) >20 × 10^9^/L, duration of dialysis (dialysdur) >10 days, anuria >1 day, any kind of complication, and antihypertensive treatment (btdur) for more than one day, were considered as severe (Table 1).

**Table 1.** Clinical outcomes of 54 patients with STEC-HUS

Clinical parameter	Severe	Mild	No. of severe/all patients* (%)
HUS episode			
Hemoglobin (g/L)	≤65	>65	17/54 (31.48)
Leukocytes (×10^9^/L)	>20	≤20	28/54 (51.85)
Duration of anuria (day)	≥1	< 1	29/53 (54.72)
Any kind of complication during the HUS episode	Yes	No	23/54 (42.59)
Duration of dialysis (days)	≥10	<10	26/53 (49.06)
Duration of antihypertensive treatment (day)	≥1	<1	17/50 (34.00)
Follow-up			
Estimated glomerular filtration rate (ml/min/m^2^ body surface area) at follow-up	<90	≥90	6/42 (14.29)
Hypertension or antihypertensive treatment at follow-up	Yes	No	4/43 (9.30)
Albuminuria at follow-up	Yes	No	14/40 (35.00)
Some renal sequelae at follow-up	Yes	No	18/44 (40.91)

*Number of patients with available corresponding clinical information.

The duration of the follow-up was continued 4 years after the acute phase of the disease. We examined three follow-up parameters: creatinine, cystatin C and estimated glomerular filtration rate (eGFR) [34]. eGFR < 90 ml/min/m^2^ (body surface area) was defined as decreased and therefore a sign of severe sequelae. The presence of hypertension or antihypertensive treatment (HT) and albuminuria (Alb) at follow-up were also defined as signs of severe sequelae. Renal sequelae (any kind of kidney-related abnormality at follow-up) was defined as the presence of at least one of the above parameters.

### DNA extraction and whole-genome sequencing

Bacterial DNA was obtained from overnight cultures on blood agar plates by EZ1 DNA Tissue Kit on EZ1 instrument (Qiagen, Hilden, Germany) according to the manufacturer’s instructions for Gram-negative bacteria. The library was prepared using Nextera DNA Flex Library Prep kit (Illumina). For the 33 isolates from Gothenburg, library was pair-end (2×100 bp) sequenced to average 2 M read pairs on HiSeq 2500 (Illumina) platform at SciLifeLab (Stockholm, Sweden). For the 21 isolates from Stockholm, library was pair-end (2×150 bp) sequenced to minimum 3 M read pairs on NovaSeq 6000 (Illumina) at SciLifeLab. Base-calling and demultiplexing were done using bcl2fastq v2.20.0.422, without allowing any mismatch in the index sequence. The sequencing reads were *de novo* assembled with SKESA (version 2.3.0) [35]. The draft genome sequences were annotated with Prokka (version 1.14.5) [36].

### Determination of stx subtypes, virulence factors, serotypes, sequence types, and O157:H7 clade 8 strains

The *stx* subtypes of STEC isolates were determined by ABRicae version 0.8.10 (https://github.com/tseemann/abricate) with default parameter. Briefly, an in-house *stx* subtyping database was created with ABRicate by including representative nucleotide sequences of all identified *stx*_1_ and *stx*_2_ subtypes. The assemblies were then used to search against the *stx* subtyping database. The assemblies were compared against VFDB database (http://www.mgc.ac.cn/VFs/) for virulence factors using ABRICATE 0.8.10 using the following parameters: coverage ≥75%, identity ≥80%. Serotype was determined by comparing assemblies to the SerotypeFinder database (DTU, Denmark) (http://www.genomicepidemiology.org/) using BLAST+ v2.2.3. Multilocus sequence typing (MLST) was conducted *in silico* using the on-line tool provided by the Warwick *E. coli* MLST scheme website (https://enterobase.warwick.ac.uk/species/ecoli/allele_st_search).

The clade 8 status of O157:H7 strains was determined using an *in silico*-PCR approach, which was based on the study from Manning *et al.* [6], an SNP in the ECs2357 gene was used for clade 8 identification in the *in silico*-PCR approach as described previously [37].

### Whole-genome phylogenetic analysis

To assess the phylogenetic relationship of HUS-STEC strains in this study, an *ad hoc* whole-genome multilocus typing (wgMLST) analysis was performed using fast-GeP (version 1.0.2) [38]. The draft genomes of 54 HUS-STEC strains were analyzed with draft genomes of 30 non-HUS STEC strains from Sweden that were processed using same pipeline as recently described [39]. The 30 non-HUS STEC representative strains included 10 STEC isolates from bloody diarrheal (BD) patients, 10 from non-bloody diarrheal (NBD) patients, and 10 from individuals without diarrhea (ND) (Table S1). The complete genome sequence of strain Sakai (Acc. NC_002695) was used as a reference. The whole-genome phylogeny was inferred from the concatenated sequences of the loci shared by all genome sequences, which was found in the *ad hoc* wgMLST analysis. All the regions with elevated densities of base substitutions were eliminated and phylogenetic relationships were generated by Gubbins (version 2.4.1) [40].

### Ethics statement

The study was approved by the regional ethics committees in Gothenburg (2015/335-15) and Stockholm (2020–02338), Sweden, respectively.

### Statistical Analysis

χ^2^ and Fisher’s exact test were used to analyze if specific virulence genes of HUS-STEC isolates were significantly related to clinical outcomes of HUS patients, and to determine the associations between bacterial genetic factors of HUS-STEC isolates, using Statistica12 (StatSoft, Inc. Tibco). A *p*-value below 0.05 was regarded as statistically significant.

### Data deposition

GenBank accession numbers of the 54 HUS-STEC genomes in this study are shown in Table S1.

## Results

### Molecular characterization of the HUS-associated STEC strains

**Figure 1.**
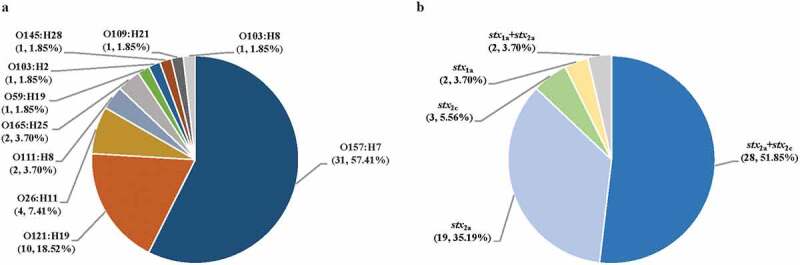
**Distribution of serotypes** (a) **and *stx* subtypes** (b) **in 54 HUS-STEC strains** The number and percentage of strains belonging to corresponding serotypes and *stx* subtypes is shown in parentheses (number, percentage)

Fifty-four HUS-STEC strains were included in the study, among which, O157:H7 was the most predominant serotype accounting for 57% (31/54) of strains. Five of “big six” non-O157 serogroups [41] were identified, namely, O121 (10/54; 19%), O26 (4/54; 7%), O111 (2/54; 4%), O103 (2/54; 4%), O145 (1/54; 2%). Notably, some non-predominant serotypes such as O165:H25 (2/54; 4%), O59:H17 (1/54; 2%), and O109:H21 (1/54; 2%) were also identified among HUS-STEC strains (Figure 1a). Five different *stx* subtypes were identified, the combination of *stx*_2a_ and *stx*_2c_ was the most common subtype, which was present in 52% (28/54) of all strains, and 87% (27/31) of O157:H7 strains, followed by *stx*_2a_ (19/54; 35%), *stx*_2c_ (3/54; 6%), *stx*_1a_ (2/54; 4%), *stx*_1a_+*stx*_2a_ (2/54; 4%) (Figure 1b). Ten MLST sequence types (STs) were assigned in 54 HUS-STEC strains. Isolates with the same serotype (O157:H7, O121: H19, O165: H25) were assigned as same ST (i.e., ST11, ST655, ST119, respectively) (Table S2). Thirty out of 31 O157:H7 strains belonged to clade 8 strains.

Serotype O157:H7 was found to be associated with *stx*_2a_+*stx*_2c_ (p < 0.01). Correspondingly, *stx*_2a_+*stx*_2c_ was associated with ST11 (p < 0.01). O165: H25 serotype showed a link with ST119 (p = 0.001).

### Clinical characterization of HUS patients

Twenty-nine out of 53 (55%) HUS patients had anuria for more than one day; this data was unavailable for one HUS patient. Twenty-six out of 53 (49%) patients needed dialysis for more than 10 days, this information was missing for one patient. Twenty-eight (28/54; 52%) patients had leukocytosis (leukocytes >20 ×10^9^/L). Twenty-three (23/54; 43%) patients developed complications during the HUS episode. Follow-up data was available for 44 out of 54 patients, among which, 18 (41%) developed renal sequelae. Detailed information of the hemoglobin levels, the presence of hypertension, duration of antihypertensive treatment, either eGFR were presented in Table 1. The prevalence of renal sequelae at follow-up, either eGFR <90 mL/min/m^2^, hypertension and albuminuria, was significantly associated with O157:H7 serotype, the presence of *stx*_2a_+*stx*_2c,_ and ST11 (Table 2).

**Table 2.** Prevalence of Renal sequelae^a^ in correlation to serotype, *stx* subtype and MLST

Serotype/*stx*/MLST	No. of isolates (%)		*p*-value
	no Renal sequelae^a^ (n = 26)	Renal sequelae (n = 18)	
O157:H7	12 (46.15)	14 (77.78)	0.036*
*stx*_2a_+*stx*_2c_	9 (34.62)	13 (72.22)	0.014*
MLST11	12 (46.15)	14 (77.78)	0.036*

^a^Renal sequelae: Any kind of kidney-related abnormality at follow-up (eGFR/HT/Alb).

* Statistically significant difference.

### Virulence factors in correlation to clinical symptoms

We identified 588 virulence factors (VFs) in 54 HUS-STEC isolates, among which 59 genes were statistically associated with the prevalence of renal sequelae at follow-up, while *cfaA* gene was negatively associated with kidney-related pathology. These virulence genes can be classified into several groups based on their functions: type III secretion system effectors (*espX2, espX3, espY2, nleB2, nleC*), type II secretion proteins (*etpD, etpE, etpG, etpH, etpI, etpJ, etpK, etpL, etpM, etpN*), chaperones (*cesAB, cesL, escE, escG*), hypothetical proteins and other factors (Table S3).

**Figure 2.**
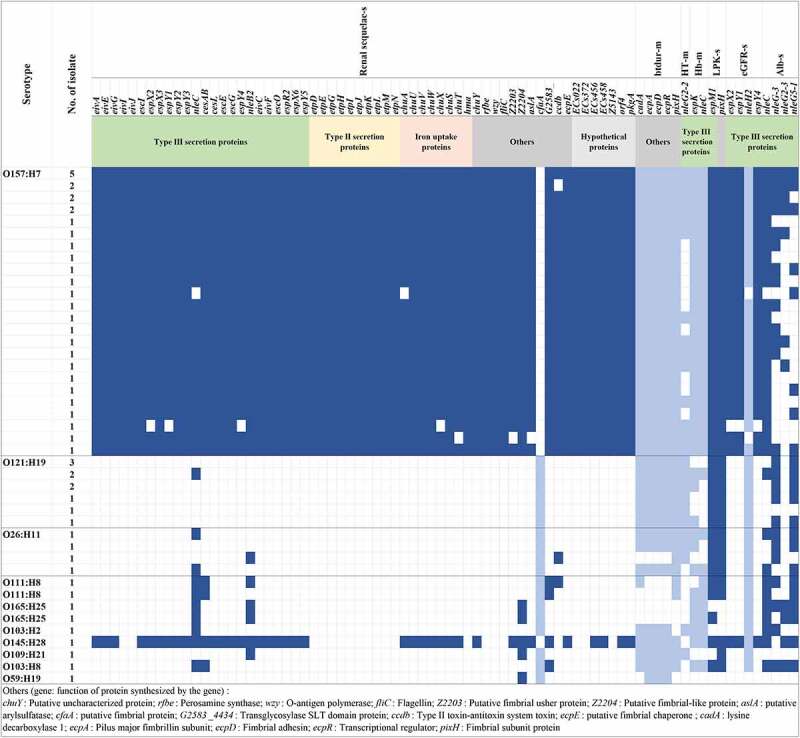
**Distribution of virulence factor genes significantly related to clinical symptoms in different serotypes** Virulence factor genes significantly associated with different clinical symptoms are shown on the top. The functions of genes are indicated below the genes. Serotypes, number of isolate with the same genes spectrum are indicated on the left. Virulence factor genes associated with severe (s) clinical symptoms are shown in dark blue, those related to mild (m) symptoms are shown in light blue, blank box means the virulence factor gene was absent. Renal sequelae: any kind of kidney-related abnormality at follow up (eGFR/HT/Alb); btdur: duration of antihypertensive treatment; HT: hypertension or antihypertensive treatment at follow up; Hb: hemoglobin; LPK: leukocytes; eGFR: estimated glomerular filtration rate; Alb: Albuminuria at follow-up

The presence of virulence genes *cadA, ecpA, ecpD, ecpR*, and *pixH* were negatively associated with antihypertensive treatment (Table S4). *nleG2-2* gene was statistically significantly associated with a mild clinical outcome with no hypertension or antihypertensive treatment at follow-up (p = 0.019). *espK* and *nleC* were associated with mild anemia (Hb > 65 g/L), with a *p*-value of 0.021 and 0.014, respectively. *espM1* and *pixH* were associated with high leukocytes level above 20×10^9^/L (p = 0.047). Interestingly, *espX2, espY1* and *espY4* were associated with decreased eGFR (<90 ml/min/m^2^) at follow-up (p = 0.029), while *nleH2* was related to normal eGFR (≥90 ml/min/m^2^) (p = 0.049). *nleG* homologs *nleG-3, nleG2-3*, and *nleG5-1*, as well as *nleC*, were associated with the presence of albuminuria at follow-up (Table S5). Virulence factors related to renal sequelae at the follow-up were significantly more prevalent in O157:H7 strains. In contrast, those associated with mild symptoms such as no hypertension or antihypertensive treatment and higher hemoglobin were relatively evenly distributed in strains of all serotypes (Figure 2).

### Phylogenomic comparison of HUS associated STEC and non-HUS STEC

**Figure 3.**
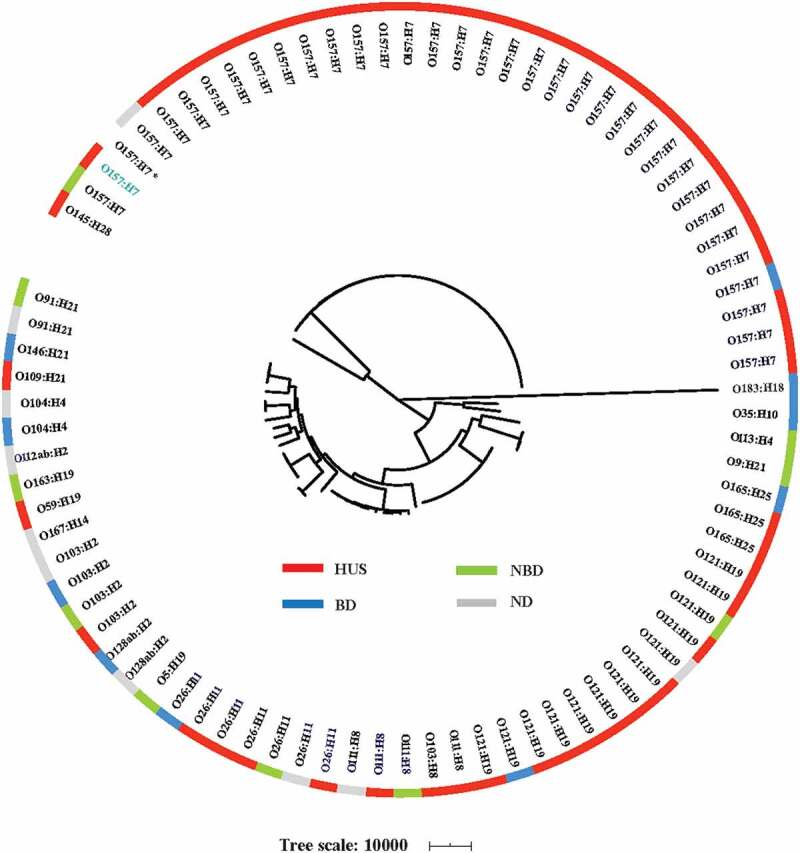
**Whole-genome phylogeny of STEC strains** Circular representation of the Gubbins tree generated from the concatenated sequences of the shared loci that found in the wgMLST analysis. Gubbins tree was annotated with strain classification using iTOL. The outer circle shows the clinical trait as indicated. One non-clade 8 O157 strain is shown in blue. Reference O157:H7 strain Sakai is indicated with an asterisk. HUS: hemolytic uremic syndrome; BD: bloody diarrhea; NBD: non-bloody diarrhea; ND: no diarrhea

The whole-genome phylogeny was used to accurately determine relationships of HUS-STEC strains, together with reference non-HUS STEC strains. In total, 2,846 shared loci were found in 85 STEC genomes used in this study, comprising of 54 HUS-STEC strains, 30 non-HUS strains and one strain Sakai as reference. HUS-STEC isolates scattered throughout the phylogenetic tree, with most strains being clustered in O157:H7 clade. Surprisingly, all O157:H7 strains with one exception belonged to highly virulent clade 8 (Figure 3). No distinct cluster was observed among strains from non-HUS including bloody or non-bloody diarrheal cases, and individuals without diarrhea. Isolates with the same serotype were grouped together (Figure 3).

## Discussion

This is the first genomic study on clinical HUS-associated STEC isolates systematically collected over a long period in Sweden. The incidence of HUS among STEC-infected individuals is increasing, with current estimation of 5 to 15 cases per 100 STEC-patients, or even higher [42–44]. A microbiologic criterion released by Europe legislation for specific STEC serogroups, highlighted O157, O26, O103, O111, O145 and O104 as the most common cause for HUS in European Union [45]. An earlier study analyzed a large collection of HUS-associated STEC isolates in Germany, demonstrating that O157:H7/H^−^ (67.7%) were the most predominant serotypes among 524 STEC isolates from HUS patients [46]. Consistently, our study showed that O157:H7 was the main STEC serotype (57%) associated with HUS cases in Sweden, which was also in line with a recent study indicating that O157:H7 caused the majority of HUS cases in Finland [26]. Although the proportion of non-O157 STEC infection (mainly caused by O26, O45, O103, O111, O121, and O145 serogroups) has increased in recent years [47,48], O157:H7 still remains as the predominant serotype to cause severe clinical outcomes such as HUS. Among non-O157 serotypes, O121:H19 was the most common serotype (19%) in this study, which was different from the German study [46], where O26:H11/H^−^ (42.6%) was the most predominant serotype among 169 non-O157 HUS-associated STEC strains, followed by O145:H28/H – (18.9%), O111:H8/H – (8.3%), and O103:H2/H – (8.3%). These results indicated that O157:H7 remains as main cause of HUS globally, while the predominant non-O157 STEC serotypes causing severe diseases varied among different countries. It has been reported that Stx genotype rather than the amount of Stx or the cytotoxicity correlates with the appearance of the HUS, i.e., the *stx*_2a_ and *stx*_2c_ were associated with high virulence and the ability to cause HUS [18]. In line with earlier studies, we observed that *stx* subtype *stx*_2a_+*stx*_2c_ (52%) and MLST type ST11 (57%) accounted for the largest proportion among 54 HUS-STEC strains in the present study, which all belonged to O157:H7 serotype, with one exception. Among the HUSEC collection in Germany, *stx*_2a_ is the most predominant *stx* subtype [46], similar with a Finnish study, indicating that *stx*_2a_ was one risk factor for HUS in STEC-positive children [26]. Notably, we found that O157:H7, *stx*_2a_+*stx*_2c_, and ST11 were associated with renal sequelae at follow-up, indicating that O157:H7 is highly associated with development of long-term sequelae.

STEC pathogenesis is complex as various bacterial and host factors interact with each other, and contribute jointly to disease progression. The association between bacterial factors and disease severity has not been well defined in HUS cases, as most studies mainly focus on several key virulence factors. In this study, we attempt to identify the discriminative genetic features of STEC strains associated with severe outcomes of HUS. We found that *espM1* and *pixH* were associated with higher leukocytes level in acute phase, *espX2, espY1* and *espY4* were associated with decreased eGFR at follow-up, and *nleC, nleG-3, nleG2-3*, as well as *nleG5-1* were associated with follow-up albuminuria. Approximately sixty virulence factors, including *chuA, eivA, etpD, espX* and their family genes, as well as *nleB2* and *nleC*, were found to be associated with renal sequelae at follow-up. The functions of some virulence factor genes remain unclear. Notably, some virulence genes related to severe outcomes play a significant role in the STEC pathogenesis. For instance, *nleB*, the encoded protein can suppress host cell death and inflammation [49], which was shown to be highly associated with enhanced virulence for humans [50]; *nleC*, which could repress NF-κB and MAPK signaling [51]; *espM*, which inhibits pedestal formation and disrupts the architecture of a polarized epithelial monolayer [52]. Most of the unfavorable-outcome related-virulence genes were only found in O157:H7 strains, while those associated with better outcome were distributed among all serotypes, indicating that O157:H7 pose higher risk for severe renal sequelae of HUS. Hence, this study suggests that a subset of the genes associated with severe outcomes could be included in the STEC diagnostics, in addition to key virulence genes, e.g., *stx, eae*. Further studies are warranted to detect the expression level and function of these genes in correlation to disease severity.

The whole-genome phylogeny indicated high diversity of STEC strains. HUS-STEC strains scattered throughout the phylogenetic tree, with most strains being clustered in O157:H7 clade. O157:H7 strains were classified into nine different clades, among which, clade 8 strains were reported to produce higher level of Stx2 [53,54], which might partly explain why these strains cause severe disease and HUS. We found that all O157 strains with one exception in this study belonged to clade 8, consistent with a previous study indicated that HUS patients were more frequently associated with strains from clade 8 [6]. However, our study could not clearly distinguish between HUS-associated and non-HUS STEC strains on the genomic level, HUS-STEC strains scattered throughout the O157 clade and non-O157 clades on the whole genome phylogenetic tree. This was in agreement with an earlier Norwegian study [55]. The present study implies that other factors than bacterial genetic factors, for instance, genes expression level, host-related factors, and bacteria-host interactions might play a joint role in STEC pathogenesis, which is valuable future work.

In conclusion, the current study confirms that O157:H7 serotype is the main cause of HUS, while the predominate non-O157 STEC serotypes associated with severe disease vary among countries. *stx* subtype *stx*_2a_+*stx*_2c_ and several other virulence factors genes were found to be associated with severe clinical outcomes. In addition, rapid diagnostic targeting of these genes directly on the fecal samples may contribute to improved care and infection control measures ultimately leading to less patients suffering from severe STEC disease.

## Supplementary Material

Supplemental Material
