# Supplementary material for: Whole-genome characterization of hemolytic uremic syndrome-causing Shiga toxin-producing Escherichia coli in Sweden
Source: Virulence. 2021 May 3;12(1):1296–305. doi: 10.1080/21505594.2021.1922010 (PMC8096335; doi:10.1080/21505594.2021.1922010)
Supplement: Supplemental Material [file KVIR_A_1922010_SM7083.zip › Document.rtf]

Supplementary Materials
Table S1. Metadata of 54 HUS-causing STEC isolates (.xlsx)
Table S2. Serotypes, sequence types and stx subtypes in 54 STEC isolates (.doc)
Table S3. Association between virulence genes and renal sequelae (.xlsx)
Table S4. Association between virulence genes and duration of antihypertensive treatment (.doc)
Table S5. Association between virulence genes and albuminuria at follow-up (.doc)
