# Supplementary material for: Whole-genome characterization of hemolytic uremic syndrome-causing Shiga toxin-producing Escherichia coli in Sweden
Source: Virulence. 2021 May 3;12(1):1296–305. doi: 10.1080/21505594.2021.1922010 (PMC8096335; doi:10.1080/21505594.2021.1922010)
Supplement: Supplemental Material [file KVIR_A_1922010_SM7083.zip › revised TABLE S2,S4,S5clean.doc]

**Table S2.** Serotypes, sequence types and *stx* subtypes in 54 STEC isolates

| **Serotypes** | **No. isolates (%)** | **ST (No. of isolates)** | ***stx* (No. of isolates)** |
| --- | --- | --- | --- |
| O157:H7 | 31 (57.41) | ST11 (31) | *stx*_2a_+*stx*_2c_ (27), *stx*_2c_ (2), *stx*_2a_ (2) |
| O121:H19 | 10 (18.52) | ST655 (10) | *stx*_2a_ (9), *stx*_1a_+_2a_ (1) |
| O26:H11 | 4 (7.41) | ST21 (3), ST29 (1) | *stx*_2a_ (2), *stx*_1a_ (1), *stx*_2c_ (1) |
| O111:H8 | 2 (3.70) | ST327 (1), ST16 (1) | *stx*_1a_+*stx*_2a_ (1), *stx*_2a_ (1) |
| O165:H25 | 2 (3.70) | ST119 (2) | *stx*_2a_ (1), *stx*_2a_+*stx*_2c_ (1) |
| O59:H19 | 1 (1.85) | ST1136 (1) | *stx*_2a_ (1) |
| O103:H2 | 1 (1.85) | ST386 (1) | *stx*_1a_ (1) |
| O145:H28 | 1 (1.85) | ST32 (1) | *stx*_2a_ (1) |
| O109:H21 | 1 (1.85) | ST40 (1) | *stx*_2a_ (1) |
| O103:H8 | 1 (1.85) | ST2836 (1) | *stx*_2a_ (1) |
| Total | 54 | | |

**Table S4.** Association between virulence factor genes and duration of antihypertensive treatment

| **Gene** | **Severe btdur^a^ (n=17)** | **Mild btdur (n=33)** | ***p*-value** |
| --- | --- | --- | --- |
|  | **No. positive (prevalence %)** | **No. positive (prevalence %)** |  |
| *cadA* | 13 (76.47) | 32 (96.97) | 0.04^*^ |
| *ecpA* | 13 (76.47) | 32 (96.97) | 0.04^*^ |
| *ecpD* | 13 (76.47) | 32 (96.97) | 0.04^*^ |
| *ecpR* | 13 (76.47) | 32 (96.97) | 0.04^*^ |
| *pixH* | 13 (76.47) | 33 (100.00) | 0.01^*^ |

^a^: btdur: duration of antihypertensive treatment, ﹤1day: Mild; ≥1 day: Severe

^*^ Statistically significant difference.

**Table S5.** Association between virulence factor genes and albuminuria at follow-up

| **Gene** | **Severe Alb^a^ (n=14)** | **Mild Alb (n=26)** | ***p*-value** |
| --- | --- | --- | --- |
|  | **No. positive (prevalence %)** | **No. positive (prevalence %)** |  |
| *nleC* | 14 (100.00) | 18 (69.23) | 0.034^*^ |
| *nleG-3* | 11 (78.57) | 12 (46.15) | 0.048^*^ |
| *nleG2-3* | 8 (57.14) | 5 (19.23) | 0.031^*^ |
| *nleG5-1* | 10 (71.43) | 10 (38.46) | 0.047^*^ |

^a^: Alb: albuminuria at follow-up, no: Mild; yes: Severe

^*^ Statistically significant difference.
